# A patient-centric analysis to identify key influences in allergic rhinitis management

**DOI:** 10.1038/s41533-018-0100-z

**Published:** 2018-09-13

**Authors:** Biljana Cvetkovski, Rachel Tan, Vicky Kritikos, Kwok Yan, Elizabeth Azzi, Pamela Srour, Sinthia Bosnic-Anticevich

**Affiliations:** 1 Woolcock Institute of Medical Research, The University of Sydney, 431 Glebe Point Road, Glebe, Sydney, NSW 2037 Australia; 2 Department of Respiratory Medicine, Royal Prince Alfred Hospital, 50 Missenden Road, Camperdown, Sydney, NSW 2050 Australia; 3 Central Sydney Local Area Health District, Level 11, KGV Building, Missenden Road, Camperdown, Sydney, NSW 2050 Australia

## Abstract

Allergic rhinitis (AR) is increasingly becoming a patient self-managed disease. Just under 70% of patients purchasing pharmacotherapy self-select their treatment with no health-care professional intervention often resulting in poor choices, leading to suboptimal management and increased burden of AR on the individual and the community. However, no decision is made without external, influencing forces. This study aims to determine the key influences driving patients’ decision-making around AR management. To accomplish this aim, we utilised a social network theory framework to map the patient’s AR network and identify the strength of the influences within this network. Adults who reported having AR were interviewed and completed an AR network map and AR severity and quality of life questionnaires. Forty one people with AR completed the study. The AR networks of the participants had a range of 1–11 influences (alters), with an average number of 4 and a median of 5. The larger the impact of AR on their quality of life, the greater the number of alters within their network. The three most commonly identified alters were, general practitioners, pharmacists and the participants’ ‘own experience’. The strength of the influence of health-care professionals (HCPs) was varied. The proportion of HCPs within the AR network increased as the impact of AR on their quality of life increased. By mapping the AR network, this study demonstrated that there are multiple influences behind patient’s decisions regarding AR management but the role of the HCP cannot be dismissed.

## Allergies: Understanding self-management

In-depth interviews with allergy sufferers about their treatment decisions highlights a need for healthcare professionals to help optimize allergy management. Allergic rhinitis (AR), a set of conditions including hayfever, is often poorly managed by patients, partly due to the growing availability of over-the-counter medications. However, little is known about what influences patients’ self-management decisions. Biljana Cvetkovski at the University of Sydney, Australia, and co-workers interviewed 41 adults with AR. The participants also completed quality of life questionnaires and created network maps of key influencers. Cvetkovski found that patients followed many different influences when making treatment decisions, including online resources, family, friends and healthcare professionals. Three of the key influencers were general practitioners, pharmacists and patients’ own experience. Those with severe AR had a larger, more diverse network of influences and often reported treatment fatigue.

## Introduction

Allergic rhinitis (AR) is a chronic respiratory condition that is globally increasing in prevalence and receiving worldwide recognition for the burden associated with its suboptimal control.^1,2^ The seemingly innocuous symptoms of AR, which include sneezing, rhinorrhoea, nasal congestion and watery/itchy eyes, can significantly impair an individual’s quality of sleep, concentration and ability to perform their daily activities.^3^ In socioeconomic terms, the impact of AR has been measured to be in billions of dollars.^4,5^ Further to this, the burden of poorly controlled AR extends to co-morbid conditions such as asthma, making it difficult to control where AR control has not been achieved.^6^ Despite the acknowledgement of the consequences of poor AR control and resources devoted to tackling the issue, optimal AR control continues to be elusive.^7^

There are several factors that contribute to AR being a challenging condition to manage including, miscommunication between health-care professionals (HCPs) and patients about concept of AR control, the co-existence of non-AR and the suboptimal use of medicines leading to inadequate symptom relief.^2,8–10^ Where AR management has traditionally been the domain of primary HCPs such as general practitioners (GPs) and pharmacists,^11–14^ the availability of medicines for purchase without consulting HCPs has contributed to AR management becoming patient driven.^15^ A particular factor that has significant impact on AR management is the high level of patient self-selection of medications. Recent Australian research has demonstrated that just under 70% of pharmacy customers purchased a treatment for AR symptoms by self-selecting their treatment without consulting a HCP, only 15% selecting optimal treatment for their symptoms.^15^

While it is accepted that a majority of people with AR self-manage their condition, little is understood about who or what influences people with AR in their management decisions. Exploring AR management from the perspective of the patient has revealed that patients feel confident in making their own decisions with regards to their AR treatment^16^ but has so far not identified who or what are the key influencers of these decisions, what the relative level of influence is nor whether these influences change depending on the level to which AR impacts on patients’ day-to-day living. By identifying the key influences within patients’ AR networks, we can better understand both the challenges and opportunities for patients, health-care providers and the health-care system to improve AR management.

Social network theory can help us to identify a patient’s health network and the influences within it.^17^ This approach has been used to identify key influences within the networks of people who have asthma and has given us fresh insight into the role that HCPs and family members have on asthma management.^18^ Utilising a social network approach with people with AR can help us understand who and what influences patient decisions with regards to AR management.

Therefore, the overall aim of this study was to determine the influences driving patients’ decision-making around AR management, to determine their relative degree of influence and to gain an understanding of whether networks vary depending on the impact of AR on patients’ day-to-day living (quality of life (QOL)).

## Results

Fifty seven potential participants contacted the research team and expressed an interest in participating in this research. Forty seven of these potential participants were eligible to participate and provided signed consent; 87% (41/47) completed the study and were included for further analysis (the remaining 6 did not complete all components of the study). Data saturation was achieved following the 20th participant, as no new alters were nominated with the name generation technique by the remaining participants. All eligible volunteers who responded to the initial round of recruitment were enrolled and participated. Once it was established that saturation had been obtained, no further recruitment was deemed necessary. The study population were aged between 18 and 86 years with a median age of 38 years. Sixty seven percent were female. Thirty four participants were from metropolitan Sydney and 7 were from regional areas of New South Wales. Data were collected from March 2014 to December 2014.

### AR severity and impact on QOL

AR severity is summarised in Table 1.

**Table 1:** Allergic rhinitis severity category of participants (*n* = 41)

Response to 'Do you have any of the following symptoms: itchy, runny, blocked nose or sneezing when you do not have a cold?'	AR severity category	Number of participants (%)
Never	None	1 (2.4%)
Occasionally and are of little bother	Mild intermittent	7 (17.1%)
Occasionally and are quite a bother	Moderate to severe intermittent	14 (34.1%)
Most days but are of little bother	Mild persistent	7 (17.1%)
Most days and are a lot of bother	Moderate to severe persistent	12 (29.3%)

The average score for the mini-RQLQ questionnaire was 2.6 (range 0–5). The number of participants in each category of AR QOL impairment was: 1 (2.4%) in QOL_ZERO_ (score 0), 20 (48.8%) in QOL_MILD_ (score >0–2), 12 (29.3%) in QOL_MOD_ (score 3–4), and 8 (19.5%) in QOL_SEV_ (score 5–6).

### Name generation

The participants nominated GPs; pharmacists; allergists/immunologists; respiratory specialists; ear, nose and throat specialists; practice nurses; alternative therapists; parents; partners; siblings; friends/colleagues; media; internet; their own experience; dermatologists; neurologists; optometrists and opthalmologists as individuals or resources who/that have an influence on their AR management decision-making. Dermatologists, neurologists, optometrists and opthalmologists appear on the map under the category ‘other HCPs’. Parents and partners were combined as one category and siblings are represented in the category ‘family’.

### AR network: maps and bar charts

**Fig. 1:**
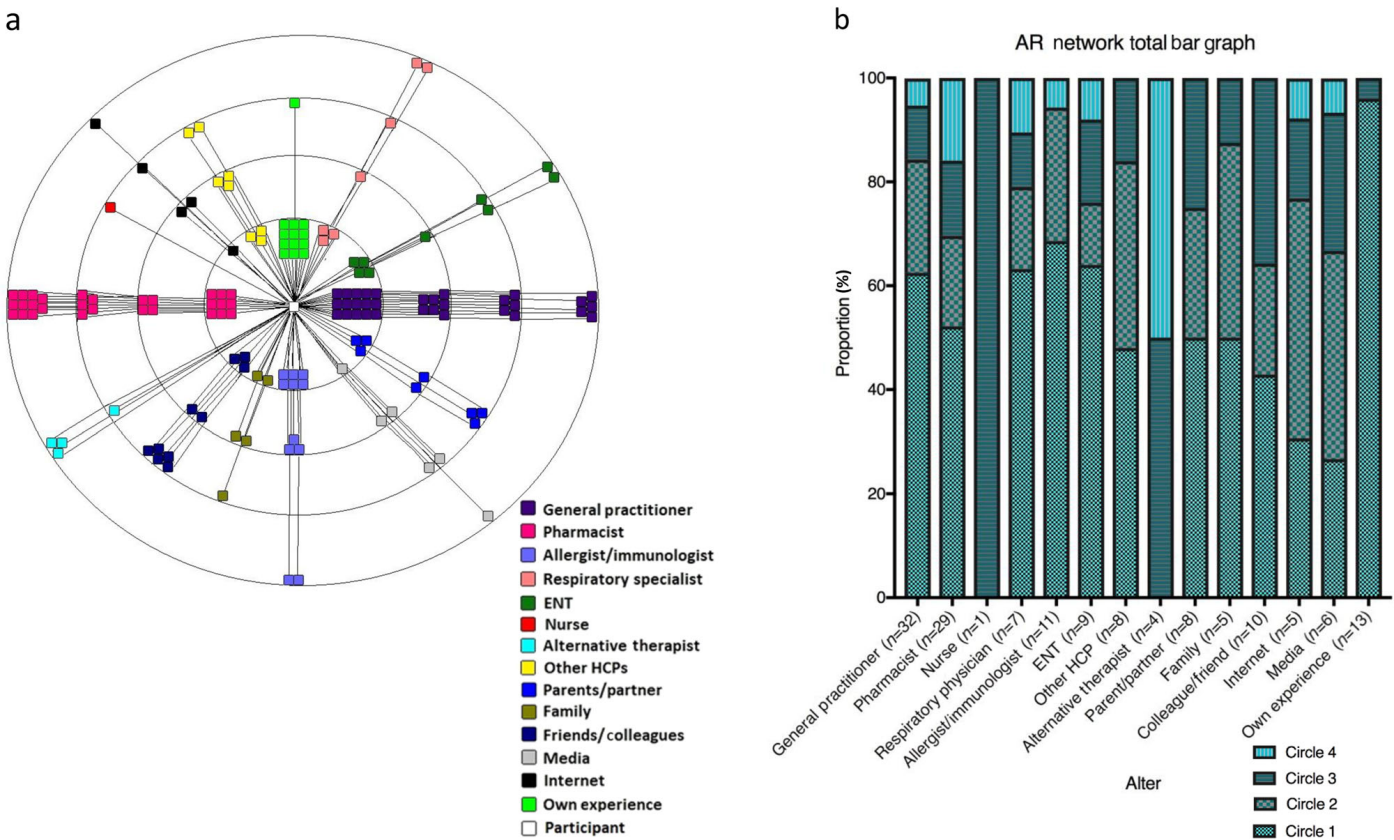
AR network total map (**a**) and bar chart (**b**)

The AR Network Map_TOTAL_ and bar chart is presented in Fig. 1a, b (*n* = 41). The number of alters nominated by participants ranged from 1 to 11, with an average number of 4 and a mode of 5. The three most commonly nominated *alters* within the AR Network Map_TOTAL_ were GPs, pharmacists and participants’ own experience.

GPs, pharmacist and ‘own experience’ constituted >50% of the alters in Circle One, being placed in Circle One by 15 (37%), 9 (22%), and 12 (30%) of participants, respectively. While GPs and pharmacists also featured prominently beyond Circle One, ‘own experience’ was almost entirely (except in one case) plotted within Circle One.

**Fig. 2:**
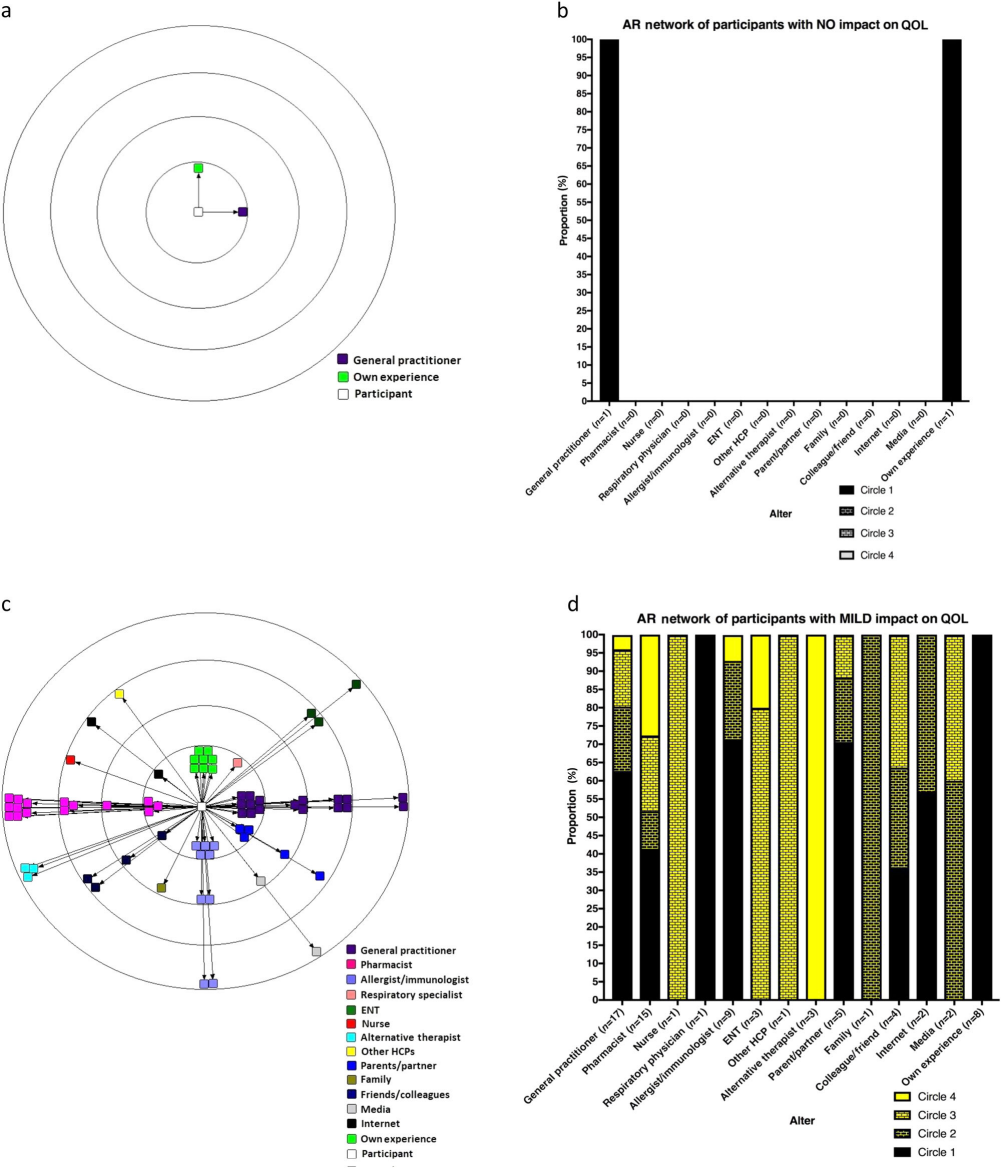
AR network map and bar chart for participants with zero (**a**, **b**) and mild (**c**, **d**) impact on quality of life

One participant reported that AR had no impact on their QOL. The AR network map and AR network bar chart for the participants who experienced zero AR impairment on their QOL (AR Network_ZERO_) are displayed in Fig. 2a, b.

Twenty participants reported AR causing mild impairment on their QOL. The AR network map and AR network bar graph for the participants who experience mild impairment due to AR on QOL (AR Network_MILD_) are displayed in Fig. 2c, d.

**Fig. 3:**
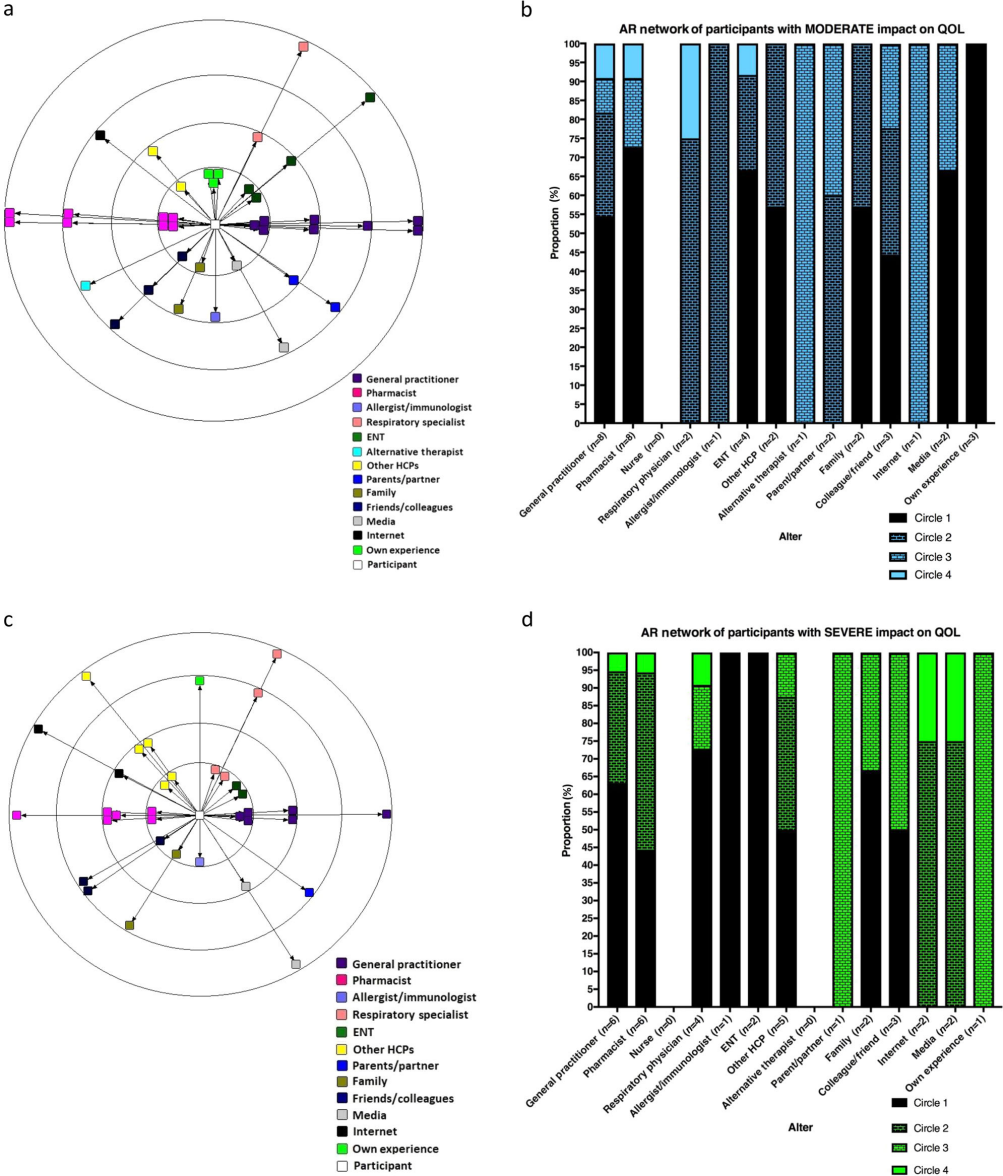
AR network map and bar chart for participants with moderate (**a**, **b**) and severe (**c**, **d**) impact on quality of life

Twelve participants reported AR causing moderate impairment on their QOL. The AR network map and AR network bar graph for participants experiencing moderate impairment due to AR on QOL (AR Network_MOD_) are displayed in Fig. 3a, b.

Eight participants reported AR causing severe impact on their QOL. The AR Network map and AR Network bar graph for participants experiencing severe impairment due to AR on QOL (AR Network_SEV_) are displayed in Fig. 3c, d.

### AR network alter density

**Fig. 4:**
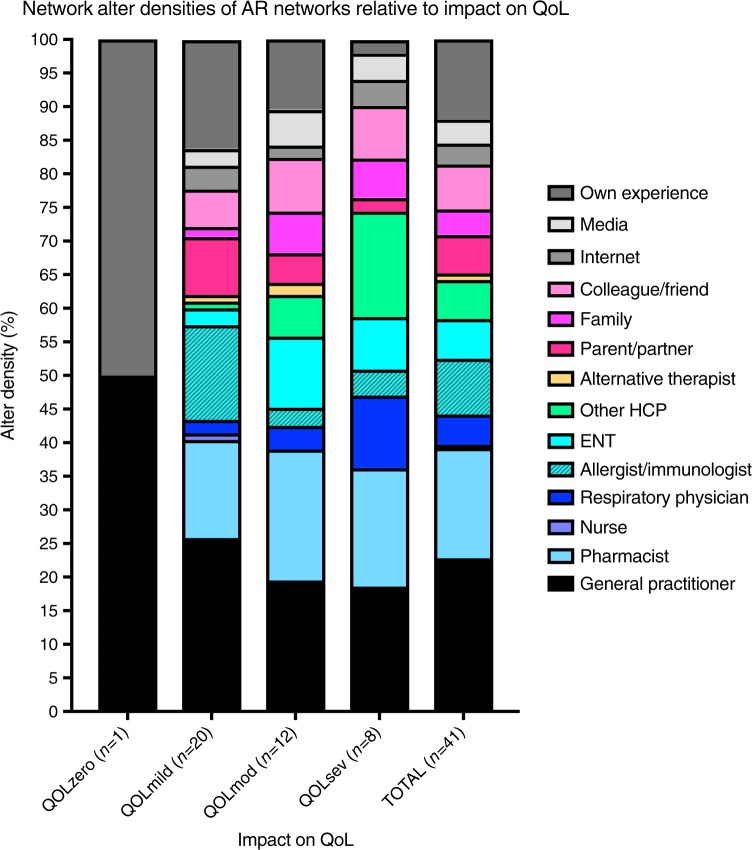
AR network alter densities relative to impact on quality of life

Figure 4 displays the AR network alter density figure for the total AR network (AR Network Alter Density_TOTAL_) and for the AR networks per QOL category (AR Network Alter DensityQOLzero, AR Network Alter Density_QOLmild_, AR Network Alter Density_QOLmod_ and AR Network Alter Density_QOLsev_, respectively).

## Discussion

This research identifies that people with AR report a wide range of influences when it comes to the management of their AR. These influences include HCPs, family and friends and information from the internet and media. When these influences are visually represented in an egocentric network map, it is evident that GPs, pharmacists and the patient’s own experience are consistently regarded as key influences on AR management. It was found that, as the impact of AR on QOL increases, the size of the patient’s AR network also increases. These findings uncover a complex network of influence for people with AR, which needs to be considered in light of the high level of self-management exemplified by this cohort of patients.

In order to address the aims of this study, a methodology embedded with social network theory was utilised. The reasons for utilising a patient-centric social network approach was based on two key empirical findings: (a) there is a high level of self-management on the part of the patient and (b) this approach was successfully used to identify novel understanding of the influence on patient self-management in asthma.^15,18^ This method enabled us focus on the patient's perspective and to specifically articulate the relative importance placed on the many and varied ‘influences’, as experienced by the patient. However, it should be acknowledged that this method represents a one-dimensional description of the patient’s AR network. To further enhance the interpretation of the data collected through the egocentric social network approach, network alter density calculations were applied. This added a deeper level of understanding to the relative importance of the different influences and adds novelty to this established method of patient-centred data collection.^18^

This research unequivocally demonstrates that the size and the composition of the AR network changes as impact of AR on QOL increases. Participants experiencing severe impact of AR on QOL have the largest networks. We often associate uncontrolled AR with an absence of HCP involvement,^15^ yet these results reveal that AR patients who experience severe impairment on their QOL identify many HCPs to be of influence within their network for AR management. The inclusion of HCPs whose specialties are not normally associated with AR management (e.g. neurologists) by this subgroup of patients suggests either the impact of AR extends beyond the respiratory system or patients feel that they are not receiving sufficient support from their primary HCP and are looking more broadly for assistance. These results clearly articulate that, in an era of treatment self-selection^15^ and patient empowerment,^16^ from the patient’s perspective HCPs remain relevant but not exclusive to the management of AR. The challenge for HCPs lies in harnessing this influence and ensuring optimal care is delivered, despite other influences, which in terms of clinical decision-making is yet unknown.

GPs appear in the AR network of the majority of patients. Following on from this, those with more severe disease consult their GPs and also have larger networks. This is an important finding in light of the fact that people with moderate-to-severe disease represent the majority of people consulting a GP,^19^ that is, it is the subgroup of people with AR who have large health networks and who are likely to be consulting the GP. In thinking about how GPs can potentially amend their approach to AR management to assist the patients with the greatest burden of disease, we need to recognise the challenges they face in managing AR. A key challenge relates to the fact that diagnosis of AR can be complicated^20–22^ and many GPs have expressed great educational need with regards to allergy management.^23^ With a limited supply per capita of allergists/immunologists (and availability of sub-lingual immunotherapy in primary care), the role of the GP in treating allergy has never been more important and upskilling our HCPs may be the solution to minimising AR-related impact on QOL in the community.^21,24^

Another HCP identified as being influential in AR management is the pharmacist. With the pharmacist, there was a high variability in the level of influence, which was noted. While it can be argued that it is the patient’s choice as to how they ‘utilise’ the pharmacist in the management of their AR, it can also be argued that, given the current availability of medicines for AR in the pharmacy, they are the first port of call for AR management. Allergic Rhinitis and its Impact on Asthma (ARIA) guidelines include pharmacists in their integrated pathway as the starting point to treatment and management, but with so many medicines available over the counter,^25,26^ we need to consider pharmacists’ skills and the resources available to them.

The inclusion of non-HCPs in the AR network demonstrates that as HCPs we must be conscious that people who are not medically trained may be influencing the decision-making of people with AR. The role of non-HCP influences have previously been identified as important in the management of asthma^27^; however, we are yet to understand the nature of their influence in AR. We need to ensure that patients with AR are themselves equipped with appropriate knowledge and understanding that their AR is more than a trivial condition. The need for patients to be better equipped in decision-making is highlighted in the high level of influence placed on patients’ ‘own experience’ in this study. Our previous findings demonstrate that a subset of people with AR believe they themselves know what works best to control their AR symptoms while experiencing treatment fatigue and disillusionment with HCP encounters.^16^ Patients need to be provided with tools to assist them and an example of such a tool is The Allergy Diary (ARIA).^26,28^ The Allergy Diary is a mobile health application that facilitates self-monitoring and collaboration with their HCPs. Further exploration of how the Allergy Diary could be implemented to address the challenges identified in this research should be undertaken in the future.

This research leaves us with several unanswered questions. Following on from this research and the identification of patient influences, it is now essential we gain an understanding of the nature of the relationships between the patient and influences and how these relationships are impacting on patient AR decision-making and one another. In particular, we must investigate patient reports of ‘own experience’ as a key influence of their AR management and whether it is a summation of previous encounters with other alters or entirely based on experimentation with over-the-counter treatments. We must also investigate what factors determine perceived level of influence and how we can use this information to optimise AR clinical outcomes. Without further information about the nature of the perceived influences and the reasons behind them, we cannot make direct connections about the nature of these influences and impact of them on AR clinical outcomes.

This study was limited by the recruitment of participants solely from New South Wales, Australia. Even though the demographics of the study population are representative of the AR population,^29^ i.e. skewed towards mild disease, a greater distribution of AR severities could potentially strengthen our understanding. A further limitation is that AR was self-reported by participants but unconfirmed (nor formal diagnosis). While this is consistent with findings in the Australian population, some of which report that 60% of individuals self-diagnose their AR^30^, ways in which to overcome this limitation should be considered in future. Variation in patient responses to AR severity and impact on QOL questionnaires (>60% of participants reported moderate-to-severe disease severity yet reported even distribution in mild versus moderate–severe categories of QOL impairment) exemplified the challenges and controversies surrounding the use of these clinical tools in AR.^31^ Having only one patient in the no impact group subsequently limited our understanding of the AR networks of people experiencing no impairment on their QOL due to AR and a larger representation of participants in this subgroup is required in future research.

In conclusion, this research has delved into the world of the patient with AR and their AR network. The most striking new knowledge generated from this research is the finding that there is a disconnect between the impact of AR on patient day-to-day living and the number of influences on which patients with AR reply. In all of this, we need to understand why the influence of HCPs is not optimising AR control and why patients feel they need so many other influences on their AR management. Understanding the effectiveness of HCPs in AR management and better supporting them to be effective is one key challenge in the real-life management of AR that needs to be addressed as a priority.

## Methods

### Study design

This study used a mixed methods approach based on the theoretical and analytical framework of social network theory as previously developed and utilised by Cheong et al.^32^ Specifically, this study used an egocentric social network framework; it focused on the network of an individual/‘ego’ (the participant) and the relationships/‘ties’ with individuals or resources/‘alters’.

### Study population

#### Inclusion criteria

The target population was people aged ≥18 years who identified themselves as having AR and able to speak English.

#### Recruitment

Advertisements were placed on the Woolcock Institute of Medical Research’s website and Facebook page. Letters were sent to individuals who were on the Woolcock volunteers’ database of patients (i.e. patients with AR who have previously either participated in research or expressed an interest in participating in research). Respondents who registered an interest were asked to contact the research team. Participation was voluntary. Written, informed consent was obtained from participants prior to commencement in the study.

#### Sample size

In an egocentric social network analysis, recruitment continues until data saturation is reached, that is no new *alters* are identified among individual health network maps.

### Data collection

In order to address the aim, the following methods were implemented during an appointment with a member of the research team:

1. *Determination of AR severity and impact on QOL*
2. *Name generator technique*
3. *Drawing of the AR network*

Participants were given the option of face-to-face or telephone interview. Those who chose to participate via the telephone were emailed/posted the study documentation for reference during the telephone call. All interviews were audio-recorded and transcribed verbatim.

**Fig. 5:**
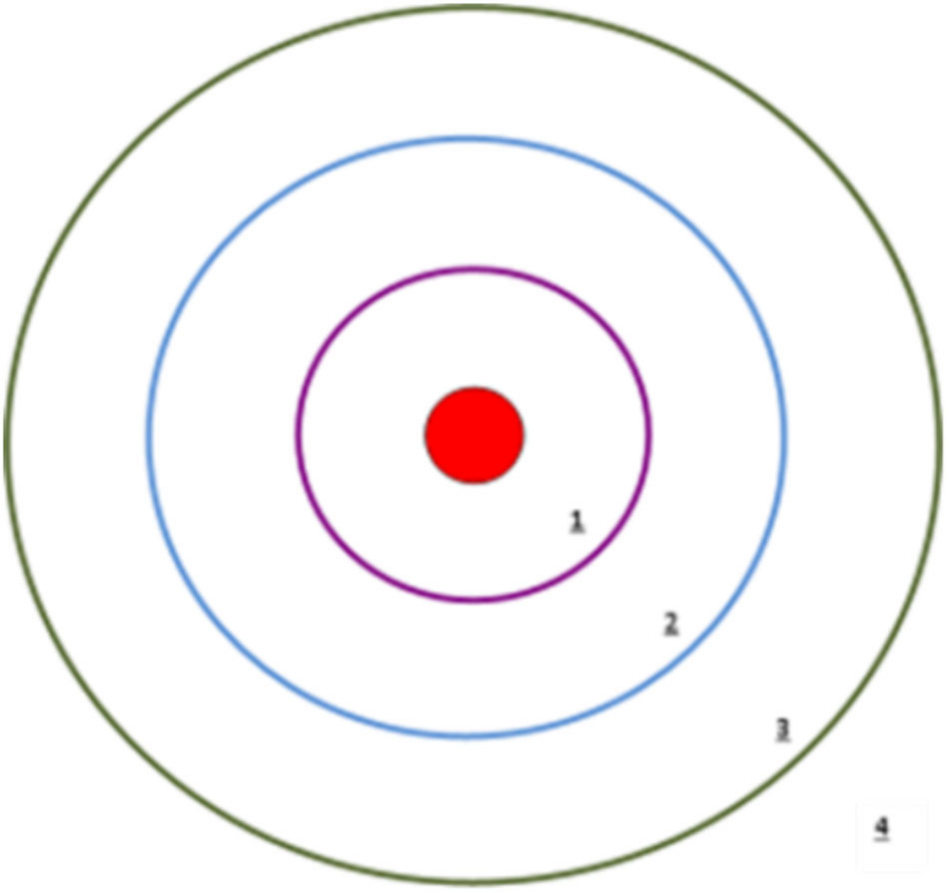
Concentric circle diagram

1. AR severity and QOL was determined by participant completed questionnaires.^30,33^
2. The name generator technique^34^ was a structured interview where the participant was asked to identify *alters* (individuals or resources) with whom they have discussed their AR within the past 5 years. A predetermined list of potential *alters*, generated from AR literature^29,35,36^ and exploratory inquiry, was used as secondary prompts in this process.
3. *Drawing the AR network*: An adapted concentric circle framework was used as the basis for the generation of each AR network map.^37^ The participant was asked to visually depict their relationship with each of their *alters* with respect to their influence on their AR management by plotting them on a concentric circle diagram (Fig. 5).

It was explained to each participant that they are represented by the red spot in the middle of the concentric circles (Fig. 5). Further to this, Circle One, closest to the centre, represented the circle on which to place the *alter*(s) who/that were most influential with regards to their AR management. Circle Four, the furthest away, represented the *alter*(s) who/that were of little influence on their AR management.

### Data analysis

#### AR severity and impact on QOL

Based on participant responses to the AR severity questionnaire, their AR severity was categorised into one of the respective five AR severity categories: no AR, mild intermittent, moderate to severe intermittent, mild persistent, and moderate to severe persistent.

Responses to the mini-RQLQ questionnaire were categorised into four groups: no impairment (QOL_ZERO_, score zero), mild impairment (QOL_MILD_, score >0–2), moderate impairment (QOL_MOD_, score 3–4), and severe impairment (QOL_SEV_, score 5–6).

#### Name generator technique

A list of alters nominated by participants was created including additional notes on whether any new alters had been identified since the previous participant’s interview.

#### AR Network maps and bar graphs

The AR network maps were drawn using NetDraw.^38,39^ Data were entered into a VNA file (format used by the NetDraw program to numerically represent alters and strength of connection) based on position of *alters* across the concentric circles, with values assigned to each *alter* corresponding to the circle in which the *alter* was plotted. An AR network map collating all participants’ individual maps was drawn (AR Network Map_TOTAL_). Individual AR network maps for all participants were drawn, as were AR network maps for subgroups of participants, i.e. for participants in each of the four QOL categories (AR Network Map_ZERO_, AR Network Map_MILD_, AR Network Map_MOD_, AR Network Map_SEV_). AR network bar graphs were drawn to quantitatively depict these AR network maps.

#### AR network alter density figure

In social network theory, network density measures the proportion of *ties* within a network relative to the total possible number of *ties*.^17^ In this egocentric network, network density principles were applied to represent the proportion (or relative influence) of *alters* within the network. In order to determine the network alter density, the strength of the tie was considered, which in an egocentric network is exemplified by the length of the tie, i.e. the placing of the *alter* on Circle 1, 2, 3 or 4. In an egocentric network, the strongest tie is that between the ‘ego’ and *alters* placed on Circle One; the weakest tie is that between the ‘ego’ and *alters* placed on Circle Four. Therefore, to capture the significance of the positioning of *alters* (and the strength of the ties), network density was calculated using the following formula:

$${\mathrm{Network}}\,{\mathrm{alter}}\,{\mathrm{density}} = \frac{{{\mathrm{Alter}}\,{\mathrm{score}}\ast }}{{{\mathrm{Sum}}\,{\mathrm{of}}\,{\mathrm{all}}\,{\mathrm{alter}}\,{\mathrm{scores}}}} \times 100$$

$${{\ast}{\mathrm{Alter}}\,{\mathrm{score}}} = n_1\mathrm{C}1x4 + n_2\mathrm{C}2x3 + n_3\mathrm{C}3x2 + n_4\mathrm{C}4x1$$

where C1 = Circle One and a weighting of 4, C2 = Circle Two and a weighting of 3, C3 = Circle Three and a weighting of 2 and C4 = Circle Four and a weighting of 1; *n*_1_C1 relates to the number of participants who placed a particular alter in Circle One; *n*_2_C2 relates to the number of participants who placed a particular alter in Circle Two, *n*_3_C3 relates to the number of participants who placed a particular alter in Circle Three, and *n*_4_C4 relates to the number of participants who placed a particular alter in Circle Four.

In this study, the network alter density enables the determination of the overall influence of *alters* for the participant population as a whole, i.e. the influence of particular individuals or resources for people with AR. The larger the value of density of the alter, the more the influence within the network.

AR network alter density was calculated for the AR Network Map_TOTAL_ and the AR Network_QOLzero_, AR Network_QOLmild_, AR Network_QOLmod_ and AR Network_QOLsev_ subgroup maps and represented in a bar graph.

This study was approved by the University of Sydney Human Research Ethics Committee and was completed in accordance with STROBE guidelines for observational research.^40^

## Electronic supplementary material

Supplementary Information

## Data Availability

The datasets generated and analysed during the current study are not publicly available because consent was not obtained from the participants to release study data.

## Electronic supplementary material

**Supplementary Information** accompanies the paper on the *npj Primary Care Respiratory Medicine* website (10.1038/s41533-018-0100-z).
