# Supplementary material for: A patient-centric analysis to identify key influences in allergic rhinitis management
Source: NPJ Prim Care Respir Med. 2018 Sep 13;28:34. doi: 10.1038/s41533-018-0100-z (PMC6137238; doi:10.1038/s41533-018-0100-z)
Supplement: Supplementary file 1 — Supplementary Information [file 41533_2018_100_MOESM1_ESM.pdf]

Supplement 1: Name generator questions

Name Generator	Primary Prompt	Secondary Prompt
Who are the people that you discuss your allergic rhinitis related matters with?	Who have you either chatted with or visited in the previous 5 years in relation to your allergic rhinitis?<br>Did you use any resources in the previous 5 years in relation to your allergic rhinitis?	Have any of the following been involved in your allergic rhinitis over the past 5 years? Doctors, pharmacists, family, friends, internet, books, etc
